# Breast cancer stigma among Indonesian women: a case study of breast cancer patients

**DOI:** 10.1186/s12905-020-00983-x

**Published:** 2020-06-03

**Authors:** Solikhah Solikhah, Ratu Matahari, Fitriana Putri Utami, Lina Handayani, Tri Ani Marwati

**Affiliations:** Faculty of Public Health, Universitas Ahmad Dahlan, Yogyakarta, 55164 Indonesia

**Keywords:** Breast cancer stigma, Indonesian women, Breast cancer screening

## Abstract

**Background:**

The stigma experienced by cancer patients stems from the association of cancer with death, as cancer is the most feared disease worldwide, especially among cancer patients and their families. The stigma regarding breast cancer screening behaviour has not been critically evaluated and is poorly understood; therefore, we aimed to analyse the stigmatization of breast cancer patients in Indonesia to reduce the morbidity and mortality of breast cancer.

**Methods:**

A qualitative study using a focus group discussion (FGD) and in-depth interviews with thematic analysis was conducted.

**Results:**

One informant experienced breast pain and kept the referral letter, in which the medical doctor advised medical treatment, to herself for 3 months due to her embarrassment. A traditional healing practice known as ‘*kerokan*’, which involves scraping of the skin, and consumption of a traditional drink were used by most informants to decrease their breast pain. Finally, most informants were diagnosed with an advanced stage of cancer when they returned to the health care facility. In addition, financial difficulties were noted as barriers to breast cancer screening in Indonesia.

**Conclusions:**

Feelings of fear and shame when diagnosed with breast cancer were reported by the informants in this study. Alternative treatment known as ‘*kerokan’* was the first treatment sought for breast cancer symptoms due to financial difficulties among breast cancer patients. Informants were diagnosed with an advanced stage of cancer after they returned to the health care facility. A better understanding of early breast cancer symptoms could motivate women to seek out breast cancer treatment.

## Background

The stigma experienced by cancer patients stems from the association of cancer with death, as cancer is the most feared disease worldwide, especially among cancer patients and their families. Breast cancer is the most common cause of cancer death among women worldwide, including in Indonesia. Additionally, the incidence of breast cancer is ranked second among all cancers, representing 1.4 million new cases around the globe [1–3]. According to the World Health Organization (2014), of all non-communicable disease deaths in Indonesia, 13% are caused by cancer. More importantly, breast cancer when first diagnosed in women in Indonesia is often at an advanced stage. According to the World Health Organization, breast cancer can be successfully treated if diagnosed at an early phase and the patient makes lifestyle changes. Numerous previous studies have revealed that most patients with cancers diagnosed at an advanced stage had a poor prognosis [4–6].

Most breast cancer deaths in developing countries such as Indonesia result from a very poor awareness of breast cancer [7], lack of biomedical treatment [8], and breast cancer stigma [9]. Breast cancer stigma and misconception of screening methods were identified as potential factors that delayed medical help-seeking behaviour in patients with breast cancer signs and symptoms [10].

Moreover, in Indonesia, as a developing country with no routine national mammography or clinical breast examination programme due to the limited number of health care facilities, most breast cancer patients are diagnosed at an advanced stage, as reported by the Health Ministry of the Republic of Indonesia in 2016. Indonesians represent many groups and cultures, and breast cancer stigma is strongly influenced by culture and beliefs. The stigma regarding breast cancer screening behaviour has not been critically evaluated and is poorly understood; therefore, we aimed to analyse the stigmatization of breast cancer patients in Indonesia to reduce the morbidity and mortality of breast cancer.

## Methods

### Study design and sample population

This study was conducted in October 2017 in Yogyakarta. Using purposive sampling, eight women with breast cancer were interviewed in the PKU Muhammadiyah Hospital in Yogyakarta. Purposive sampling was used to obtain data that were suitable for the purposes of the study by careful selection of the informants [11]. The participants were chosen with certain criteria; namely, they were selected from oncology patients at PKU Muhammadiyah Hospital, they received treatment at least 6 months before the data collection was conducted, and they agreed to participate in the study. The research and development department staff of the hospital provided the data on the outpatients that we needed. We found fifteen women with breast cancer during 2016–2017 from the list of the patients. Then, we contacted the participants by cellular phone. Five persons had died, another three persons declined to be interviewed, and one patient could not be contacted. Ultimately, six breast cancer survivors agreed to be interviewed. We also conducted interviews with two the family members of the participant: Aster 48 years old (younger sister of Lily), and Camellia, 40 years old (younger sister of Jasmine).

### Data collection

In this study, focus group discussion (FGD) and in-depth interviews were used to collect the data. FGD was used to explore patients’ perception of breast cancer, family support, and health insurance coverage for the medication [12]. The discussion provided meaningful information from other survivors as well as a support system. In-depth interviews were used to complete and integrate the information from the discussion about the role of family member support in the patient’s life. Family members completed in-depth interviews to explore their role in the quality of life of breast cancer patients.

### Data analysis

Each informant was identified in each portion of the data analysis, and all the data were managed by the researcher. All the audio-recorded interview sections were transcribed in the informants’ language (Indonesian language) by the research assistants [13], [14]. All the transcripts were checked against the recordings by the researcher, and each topic was related to the aim of the study using the research framework (health belief model). Regarding the incorporation of different information, triangulation of data sources was performed with two family members from different informants who were interviewed. Finally, all data in this study were identified and analysed based on the themes emerging from the data.

## Results

### Demographic features

Indonesian women over 30 years old with breast cancer participated in this study. Eight participants represented the primary informants in this study. All informants worked as housewives. The majority of informants were diagnosed with stage 3 breast cancer. A detailed description of the informant information is presented in Table 1.

**Table 1:** Demographic characteristics of breast cancer patients

No.	Participants (Pseudonym)	Age	Marital Status	Job	Level of Education
1.	Rose	47	Married	Housewife	Senior high school
2.	Jasmine	44	Married	Housewife	Senior high school
3.	Bougainville	57	Married	Housewife	Senior high school
4.	Orchid	51	Married	Housewife	Junior High School
5.	Daisy	43	Married	Housewife	Senior high school
6.	Lily	57	Married	Housewife	Junior High School
7.	Aster	48	Married	Housewife	Senior high school
8.	Camellia	40	Married	Housewife	Senior high school

### Early diagnosis of breast cancer

The majority of informants experienced different feelings when they were first diagnosed with breast cancer. The majority of informants reported pain in the breast and/or chest that pierced into the back and the detection of a lump in the breast. When the informants felt breast pain, they were treated with scraping, also known as ‘*kerokan’*. Pain in the breast was considered a symptom of colds. The term ‘*kerokan*’ itself describes a traditional healing method among Javanese people that involves scrapping the skin over the spine. The purpose of this treatment is to reduce the cold or pain. This finding was supported by in-depth interviews with the informants:

“... Starting in the beginning of 2016, I received examinations in the surgical ward for breast complaints and pain when standing up that reached to my back. Sometimes I felt pain in my chest, especially on the left side. It was like a heart attack…so painful. I went to the doctor and was diagnosed with breast cancer. I always take care of my health, and I often do breast self-examination (BSE). However, the pain of a lump appeared in the left breast..” (*Orchid*).

Another informant disclosed what her experiences had been since mid-2013. Her complaints were feeling soreness, as when having a cold, and finding a lump upon palpation of the breast. The informant was afraid to follow up on her health status with the doctor and felt ashamed that the doctor performed palpation. Informants preferred the use of traditional self-healing to cure their pain (reduce the pain in the breast). Traditional treatment involved the consumption of white turmeric, boiled soursop leaves, Japanese ants, and moringa leaf. After she took the traditional medication, the lump in her breast became larger, but she still did not tell her other family members.

The informant sought out medical treatment and met with a female doctor. The first doctor she met diagnosed her with breast cancer and then referred her to the hospital. The informant was diagnosed with stage 3 cancer. At the time of the referral, the informant did not go directly to the hospital and kept the referral letter for 3 months. During those 3 months, the informant experienced chest pain, a red rash, blood and mucus drainage from the nipple, and nipple peeling and wounds and used an ointment that she purchased alone at the pharmacy. The informant ultimately felt the need to visit the hospital again and ask for a referral letter to return to the health care facility. Doctors suggested that she visit the hospital promptly before the disease worsened. The doctor’s advice motivated the informant to seek treatment after 4 years of keeping her diagnosis a secret. Informants needed time to communicate with close relatives (husbands and children) if they were to undergo an operation.

“ I was afraid of bothering and saddening my family if I explained that I have breast cancer. I kept my illness a secret for several years until the doctor finally forced me to undergo breast removal surgery” (*Lily*).

### Psychology, attitude, and social support (family members and neighbours)

Most of the informants felt shocked when they were diagnosed with breast cancer. They tried to hide it from their family. Some informants also experienced feelings of acceptance of their fate, but they had concerns about the high cost of treatment. The informants felt that the national Indonesian health insurance known as *Badan Penyelenggara Jaminan Sosial Kesehatan* (*BPJS Kesehatan*) was helpful, although the financial assistance provided by the national health insurance did not completely cover all types of services. Laboratory examinations and early-stage examinations are not paid for by Indonesian health insurance. These findings were supported by the following in-depth interview:

“... I gained motivation from friends who suffer from the same pain and are even younger than me; got support from children, so I did not feel alone; close family support became closer during the pre-time and post-operation process” (*Rose*).

“… Yes I used the *BPJS Kesehatan* .. *Alhamdulillah* (read: Thank God) the *BPJS Kesehatan* helped me a lot to ease my medical expenses, although not all are free. I did several laboratory tests before the operation and I had to pay for it by myself” (*Daisy*).

The family member of the patient also said that she always supported her sister. Supporting acts for instance, were to accompany her sister to the hospital, give a massage to her sister when the pain arose, to remind her sister to take her medicine, and most importantly, to provide encouragement to improve her sister’s mental wellbeing.

“…I always accompany her to get a medication at the hospital…her husband left her, so I have to help her. I have to support her in every moment, including taking care of her two children. I am pretty sure she will be healthy” (*Aster*).

“…I know it is very hard for her…she must lose one of her breasts…as a woman I know what she felt. She lost weight; she is so thin. I understand it would be difficult to accept an unhealthy condition. Sometimes, I make jokes with her to support her, and I always remind her to take the drugs. I also said to my sister, ‘you have to be healthy for your family, your children” (*Camellia*).

### Communication between doctor and patients

Most of the research informants explained that they needed detailed information about the disease from the doctor. Four of the informants said that the doctor did not explain the disease clearly. Some informants mentioned that doctors who examined them were not communicative in conveying the patients’ health condition, and thus they felt worried about their condition. This finding is based on the results of in-depth interviews with informants as follows:

“... Doctors should provide as much information as possible about the illness and stages suffered by the patient, provide a transparent explanation of the lab results so as not to make the patient feel anxious guessing and guessing about cancer they are experiencing” (Bougainville).

“ …I did not get detailed information about my disease. The doctor just said. I have a cancer in my breast, it is the third stage, and he asked me to have surgical procedure. I was shocked….. I did not even know what kind of the disease I had. At that time, I just thought would I forfeit one side of my breast?” (Orchid).

## Discussion

Breast cancer has the second highest incidence rate across all types of cancers and ranks as the fifth highest cause of cancer deaths overall [15]. Delays in presentation and diagnosis are major determinants of breast cancer survival [16]. Perceptions towards breast cancer screening and diagnosis have an important role in the early diagnosis of breast cancer. Women who have healthy beliefs regarding breast cancer will enjoy an improved quality of life after receiving breast cancer treatment, including breast cancer screening [17]. However, the present research found that Indonesian women had negative perceptions towards breast cancer screening because of their experience of fear and shame.

Previous research also showed that the fear of discovering cancer, embarrassment, and fear of the screening procedure were among the most commonly reported personal or cultural barriers to using the screening service [18]. Other research studies found that fear of suffering from the disease received the highest score of all reasons for fear, and this fear might contribute to the reduced willingness of women to be screened and to learn of their disease [19]. Asian women are generally more private in their perceptions of their body and less receptive to revealing private parts of their body even to health personnel [17]. This explains why many informants in this study felt shame due to breast cancer screening. The feeling of shame led Indonesian women to receive a complementary alternative treatment known as ‘*kerokan’* and to consume white turmeric and Japanese ants. Complementary alternative treatment is valued, more convenient, and more widely available and affordable than modern medicines [17].

Indonesian women in this study also faced financial barriers because the national Indonesian health insurance, known as *BPJS Kesehatan*, did not completely cover all types of services. Several articles have mentioned that low income is one of the key barriers to early diagnosis. Although health insurance can help cover the costs of services, it was also noted that most women with an income are able to afford their own transportation, hence improving their accessibility to health care centres [17].

This study revealed not only the barriers that Indonesian women face regarding breast cancer screening but also how they cope with their breast cancer status. In this study, the informants received social support from family and other cancer survivors to face breast cancer, and they also gained strength through prayer. Adequate social support from close people, such as family, friends and neighbours, significantly improved the quality of life of breast cancer patients [20]. Support from peers can lead to disclosure of more personal information, the discussion of more private stories, and the exchange of more emotional support; in addition, these patients seek less help but provide more support and shift their interest from cancer diagnosis to cancer treatment [21]. These supportive resources can be emotional, physical, informational, and companionship-related [17]. Prayer may invoke a relaxation response, which in turn may positively affect their health and overall well-being [17].

We acknowledge that a limitation of this study is the sampling of informants from health care facilities. The informants may reflect certain characteristics of women who may have better health-seeking behaviour than other women. Therefore, conducting this study in the community would provide a better representation of breast cancer screening in Indonesian women.

## Conclusions

In conclusion, feelings of fear and shame related to breast cancer were identified in this study. Alternative treatment using scraping known as ‘*kerokan’* was the first treatment sought for breast cancer symptoms due to financial difficulties among patients with breast cancer. A better understanding of early breast cancer symptoms could prompt this population to seek out breast cancer treatment.

## Supplementary information

**Additional file 1.**

## Data Availability

The datasets used and/or analysed during the current study are available from the corresponding author on reasonable request.

## Abbreviations

*BPJS Kesehatan*: *Badan Penyelenggara Jaminan Sosial Kesehatan*
BSE: Breast Self-Examination
FGD: Focus Group Discussion
PKU Muhammadiyah: *Pusat Kesehatan Umum Muhammadiyah*
WHO-CIOMS: World Health Organizations - The Council for International Organizations of Medical Sciences

## References

[1] Ghoncheh M, Momenimovahed Z, Salehiniya H (2016). Epidemiology, incidence and mortality of breast cancer in Asia. Asian Pac J Cancer Prev.

[2] Ghoncheh M, Pournamdar Z, Salehiniya H (2016). Incidence and mortality and epidemiology of breast cancer in the world. Asian Pac J Cancer Prev.

[3] Youlden DR, Cramb SM, Yip CH, Baade PD (2014). Incidence and mortality of female breast cancer in the Asia-Pacific region. Cancer Biol Med.

[4] Lopes LV, Miguel F, Freitas H, Tavares A, Pangui S, Castro C (2015). Stage at presentation of breast cancer in Luanda, Angola - a retrospective study. BMC Health Serv Res.

[5] Jedy-Agba E, McCormack V, Adebamowo C, Dos-Santos-Silva I (2016). Stage at diagnosis of breast cancer in sub-Saharan Africa: a systematic review and meta-analysis. Lancet Glob Health.

[6] Vieira RA, Formenton A, Bertolini SR (2017). Breast cancer screening in Brazil. Barriers related to the health system. Rev Assoc Médica Bras.

[7] Fan L, Strasser-Weippl K, Li J-J, St Louis J, Finkelstein DM, Yu K-D (2014). Breast cancer in China. Lancet Oncol.

[8] Nelson HD, Fu R, Cantor A, Pappas M, Daeges M, Humphrey L (2016). Effectiveness of breast Cancer screening: systematic review and meta-analysis to update the 2009 U.S. preventive services task force recommendation. Ann Intern Med.

[9] Banning M, Tanzeen T (2014). Living with advanced breast cancer: perceptions of Pakistani women on life expectations and fears. Cancer Nurs.

[10] Khakbazan Z, Taghipour A, Latifnejad Roudsari R, Mohammadi E (2014). Help seeking behavior of women with self-discovered breast cancer symptoms: a meta-ethnographic synthesis of patient delay. PLoS One.

[11] Tinati R, Halford S, Carr L, Pope C (2014). Big data: methodological challenges and approaches for sociological analysis. Sociology..

[12] Anarado AN, Ezeome ER, Ofi OB, Nwaneri AC, Ogbolu Y (2017). Experiences and desired nursing assistance of women on out-patient breast cancer chemotherapy in southeastern Nigeria. Psychooncology..

[13] Halverson LR, Graham CR, Spring KJ, Drysdale JS, Henrie CR (2014). A thematic analysis of the most highly cited scholarship in the first decade of blended learning research. Internet High Educ.

[14] Braun V, Clarke V (2014). What can “thematic analysis” offer health and wellbeing researchers?. Int J Qual Stud Health Well-Being.

[15] Shah NM, Nan BLT, Hui NY, Islahudin FH, Hatah EM (2017). Knowledge and perception of breast cancer and its treatment among Malaysian women: role of religion. Trop J Pharm Res.

[16] Espina C, McKenzie F, dos Santos-Silva I (2017). Delayed presentation and diagnosis of breast cancer in African women: a systematic review. Ann Epidemiol.

[17] Khan TM, Leong JPY, Ming LC, Khan AH (2015). Association of Knowledge and Cultural Perceptions of Malaysian women with delay in diagnosis and treatment of breast Cancer: a systematic review. Asian Pac J Cancer Prev.

[18] Mamdouh HM, El-Mansy H, Kharboush IF, Ismail HM, Tawfik MM, El-Baky MA (2014). Barriers to breast cancer screening among a sample of Egyptian females. J Fam Community Med.

[19] Abu-Shammala BI, Abed Y (2017). Breast Cancer screening in relation to access barriers to health care system. Int J Sci Res.

[20] Yan B, Yang L-M, Hao L-P, Yang C, Quan L, Wang L-H (2016). Determinants of quality of life for breast Cancer patients in Shanghai, China. PLOS ONE.

[21] Zhang S, Grave E, Sklar E, Elhadad N (2017). Longitudinal analysis of discussion topics in an online breast Cancer community using convolutional neural networks. J Biomed Inform.

