# Supplementary material for: Breast cancer stigma among Indonesian women: a case study of breast cancer patients
Source: BMC Womens Health. 2020 Jun 3;20:116. doi: 10.1186/s12905-020-00983-x (PMC7268729; doi:10.1186/s12905-020-00983-x)
Supplement: Supplementary file 1 — Additional file 1. [file 12905_2020_983_MOESM1_ESM.pdf]

FOCUS GROUP DISCUSSION (FGD) INTERVIEW GUIDELINE FOR BREAST CANCER STIGMA AMONG INDONESIAN FEMALE: A CASE STUDY BREAST CANCER PATIENT.

A. **History of getting breast cancer**
   1. When were you diagnosed with breast cancer? (probing: how do you feel physically at that moment?)
   2. At what stage were you diagnosed with breast cancer for the first time?
   3. What kind of examination have you diagnosed with breast cancer?

B. **Physical** Change
   1. Are there any physical changes that you have experienced after being diagnosed with breast cancer? (probing: how does the change?)

C. **Psychological Change**
   1. How did you feel when you were first diagnosed with breast cancer? (probing: how do you deal with it?)
   2. Have you any change in behaviour or attitude after you were diagnosed with breast cancer? (probing: hopeless?), how do you handle it?

D. **Family Support**
   1. How did the family react when they found out you were diagnosed with breast cancer?
   2. What kind of family support in your treatment process?
   3. Who are your family members who fully support your healing process?

E. **Experience of using national health insurance**
   1. Do you use health insurance to fund the treatment of your illness?
   2. Does the insurance really help with medical expenses? (probing: help in what part?)
   3. Are there any perceived obstacles when using the insurance? (probing: what are the obstacles and what are the solutions?)
